# Global trends in the incidence rates of MDR and XDR tuberculosis: Findings from the global burden of disease study 2019

**DOI:** 10.3389/fphar.2023.1156249

**Published:** 2023-02-24

**Authors:** Qingting Bu, Rong Qiang, Lingyan Fang, Xiaokang Peng, Hua Zhang, Hua Cheng

**Affiliations:** ^1^ Department of Genetics, Northwest Women’s and Children’s Hospital, Xi’an, Shaanxi, China; ^2^ Department of Medical Quality Control, Yantaishi Penglai Second People’s Hospital, Yantai, Shandong, China; ^3^ Department of Infectious Diseases, Xi’an Children’s Hospital, Xi’an, Shaanxi, China; ^4^ Pediatric Intensive Care Unit, Xi’an Children’s Hospital, Xi’an, Shaanxi, China; ^5^ Department of Pharmacy, Xi’an Children’s Hospital, Xi’an, Shaanxi, China

**Keywords:** tuberculosis, incidence, trend, MDR, XDR

## Abstract

**Purpose:** The study aimed to quantify the global trends of the incidence rates of multidrug-resistant (MDR) tuberculosis (MDR-TB) and extensively drug-resistant (XDR) tuberculosis (XDR-TB).

**Methods:** Cases, age-standardized rates (ASRs), and incidence rates of MDR-TB and XDR-TB during 2010–2019 were obtained from the Global Burden of Disease Study 2019. The incidence trends of MDR-TB and XDR-TB were evaluated using the estimated annual percentage changes (EAPCs) in ASRs. The relationships among the ASRs of MDR-TB and XDR-TB, the MDR rate, the XDR rate, and socio-demographic index (SDI) were assessed using locally weighted regression and Pearson’s correlation coefficient.

**Results:** The global ASR of MDR-TB on average decreased by 1.36% (EAPC = −1.36, 95% confidence interval [CI] = −2.19 to −0.52) per year whereas that of XDR-TB was stable (EAPC = 0.69, 95% CI = −0.15–1.54) during 2010–2019. The incidence trends of MDR-TB in most regions and countries were decreasing, but those of XDR-TB were increasing. People aged 35–44 and 55–64 years had the highest incidence rates for MDR-TB and XDR-TB. The MDR and XDR rates both peaked in those aged 35–44 years. Areas with higher SDI tended to have lower ASRs of MDR-TB (*p* < 0.001, *ρ* = −0.43).

**Conclusion:** The current achievements for the incidence trends of MDR-TB and XDR-TB are insufficient. More strategies and tools need to be developed to further curb MDR-TB and XDR-TB, especially in high-risk areas and age groups, and in low SDI regions.

## Introduction

Tuberculosis is a chronic infectious disease seriously endangering human health that has become a major global public health and social problem (Kazempour Dizaji et al., 2018; World Health Organization, 2021a), with 1.3 million deaths due to TB in 2020 alone (World Health Organization, 2021a). One of the main reasons is that the drug resistance of TB continues to evolve. Standard treatment involving the two most-effective drugs (isoniazid and rifampicin) can achieve excellent cure rates for drug-sensitive patients with TB (Seung et al., 2015). In the treatment of drug-resistant TB, that of multidrug-resistant is very difficult since MDR-TB is resistant to the two most-effective first-line anti-TB drugs (isoniazid and rifampicin) (Trisakul et al., 2022). Nevertheless, extensively drug-resistant (XDR) TB, as a kind of MDR, is more concerning, and is resistant to isoniazid and rifampicin as well as all fluoroquinolone and second-line injectable drugs (World Health Organization, 2018; Lin et al., 2022; Trisakul et al., 2022). The overall cure rates of MDR-TB and XDR-TB were only 56% and 39%, respectively (World Health Organization, 2018). For more than a decade, the proportion of MDR and rifampicin-resistant patients diagnosed with TB for the first time has remained around 3%–4%, and that of patients previously treated for TB has remained at 18%–21%. There are even countries with proportions of previously treated MDR-TB cases exceeding 50% (World Health Organization, 2021a). The proportion of XDR-TB in TB is rarely reported.

According to the End TB Strategy of the World Health Organization (WHO) and the UN Sustainable Development Goals (United Nations, 2015; World Health Organization, 2015), global TB deaths must be reduced by 95% in 2035 compared with 2015. With the current data, this goal is difficult to achieve (World Health Organization, 2021a), and so it is time for urgent action to end the global TB epidemic (Pan et al., 2020a; World Health Organization, 2021a). MDR-TB and XDR-TB increase the risk of death in patients with TB and hinder the achievement of the above goal. Studying the global incidence trends of MDR-TB and XDR-TB is helpful for preventing and treating TB, and thereby reducing deaths from TB. However, there has been no systematic summary addressing this issue. The purpose of this study was therefore to determine the global incidence trends of MDR-TB and XDR-TB using the Global Burden of Disease Study (GBD) 2019 data.

## Methods

### Data source

Data sources for TB within the GBD 2019 data can be explored using the online GBD Results Tool (https://vizhub.healthdata.org/gbd-results/). The ICD-10 codes for TB are A10–A19.9, B90–B90.9, K67.3, K93.0, M49.0, and P37.0, while the ICD 9 codes are 010–019.9, 137–137.9, 138.0, 138.9, 139.9, 320.4, and 730.4–730.6. The GBD Results Tool is a data set developed and supported by the Institute for Health Metrics and Evaluation, which is an independent global health research center based at the University of Washington. This database provides epidemiological information on 369 diseases and injuries during 1990–2019 for 23 age groups; for males, females, and both sexes combined; and for 204 countries and territories that were grouped into 21 regions and 7 superregions. Previous studies have described the method of estimating TB incidence from the GBD database in detail (GBD 2019 Diseases and Injuries Collaborators, 2020; GBD 2019 Risk Factors Collaborators, 2020). Briefly, the TB data were derived from population-based surveys on tuberculin and cohort studies that examined the risk of developing active TB disease as a function of induration size. An updated systematic review was performed on the GBD 2019 which included routine surveillance and surveys reported to the WHO and the risk of MDR-TB (Mesfin et al., 2014; GBD 2019 Diseases and Injuries Collaborators, 2020). From the GBD 2019 database, we extracted the age-related number of cases and age-standardized rates (ASRs) or incidence rates during 2010–2019 globally among 5 socio-demographic index (SDI) regions, 21 geographical regions, and 204 countries and territories. The rates expressed as age-standardised are based on the GBD reference population (GBD 2017 Mortality Collaborators, 2018). In the GBD, the range of data point estimates is not expressed using 95% confidence intervals (CIs), but instead using 95% uncertainty intervals (UIs). Every estimate was calculated 1,000 times, and then the 95% UI was determined by the 25th and 97fifth value of the 1,000 values after ordering them from smallest to largest (Bu et al., 2022). We also extracted the SDI of each country and region. SDI is a compound measure of income, average years of schooling, and the fertility in each location and year in the GBD database that is used to measure socio-demographic development (Pan et al., 2020b). It is the geometric mean of the 0 to 1 index of total fertility rate under 25 years of age, average education level of the population aged 15 and over, and lagging income *per capita* (GBD 2017 Disease and Injury Incidence and Prevalence Collaborators, 2018). The location with an SDI of 0 will have a theoretical minimum level of development related to health, while the location with an SDI of 1 will have a theoretical maximum level of development. For GBD 2019, the values of SDI were multiplied by 100 on a scale of 0–100 (GBD 2019 Diseases and Injuries Collaborators, 2020). It is divided into five levels: high, middle-high, middle, low-middle, and low.

### Statistical analysis

Estimated annual percentage changes (EAPCs) of incidence rates were used to evaluate the incidence trends during 2010–2019. EAPC is a summarizing and widely used measure that assesses ASR trends over a specified time period (Hankey et al., 2000; Sun et al., 2022). Natural logarithm of regression line fitting rates were used; that is, *y* = *a* + β*x* + *e*, where *y* = ln (ASR) and *x* is the calendar year. EAPC was calculated as 100×[exp(β)–1], and its 95% CI was also obtained from the linear regression model. If EAPCs and the lower limit of the 95% CI are both > 0, then ASR is considered to have an increasing trend. In contrast, if both EAPC estimation and the upper limit of the 95% CI are < 0, ASR has a downward trend. For other values ASR is considered stable. We also assessed the relationships among ASR of MDR-TB and XDR-TB, MDR and XDR rates, and SDI using locally weighted regression and Pearson’s correlation coefficient. The MDR and XDR rates are the ratios of new MDR-TB and XDR-TB cases to new TB cases, respectively. The *p* < 0.05 was considered significant. R software (version 3.4.3) was used for the statistical analysis.

## Results

### Multidrug-resistant tuberculosis

**FIGURE 1:**
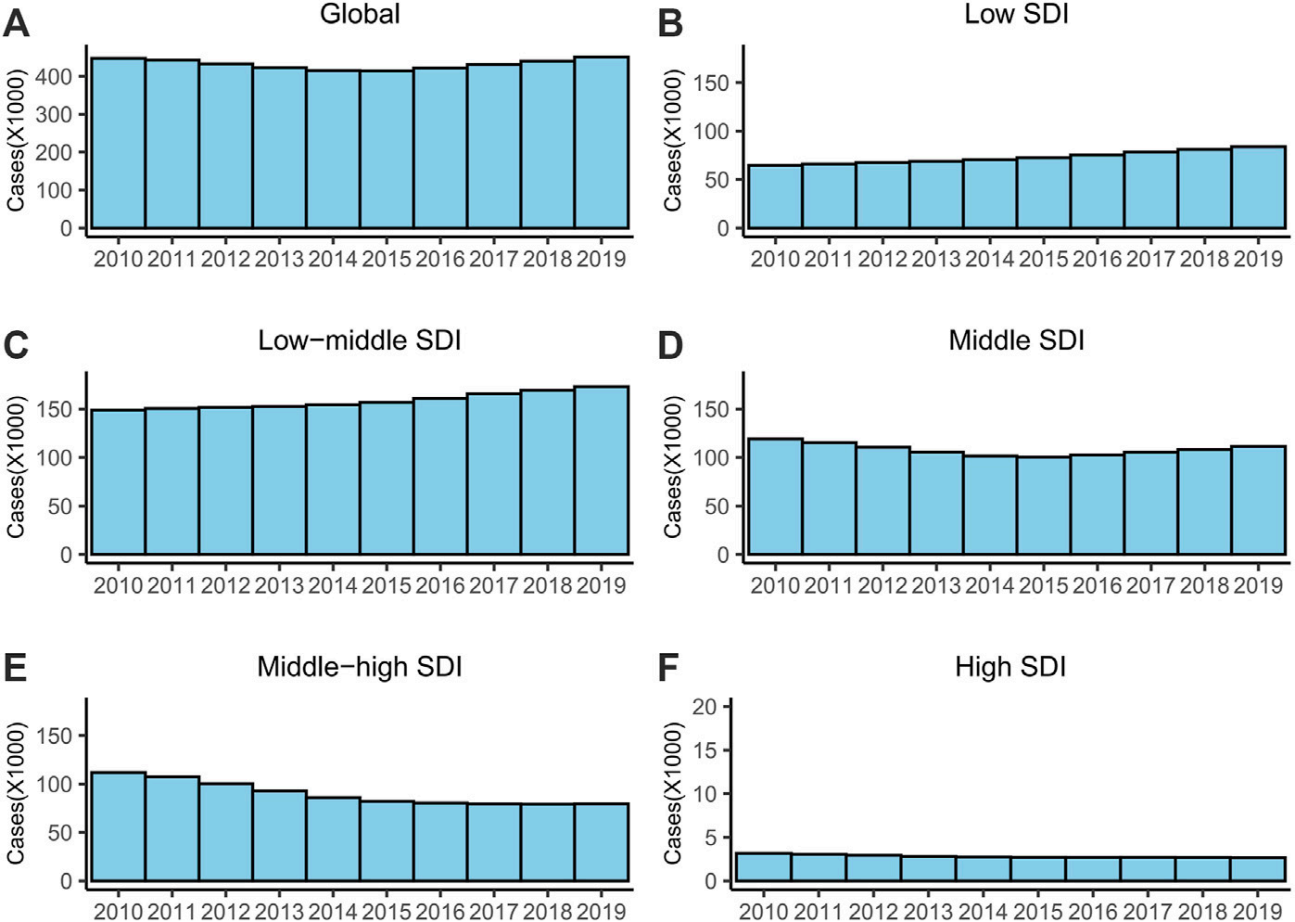
The cases of MDR-TB from 2010 to 2019. **(A)** Global, **(B)** low SDI regions, **(C)** low-middle SDI regions, **(D)** middle SDI regions, **(E)** middle-high SDI regions and **(F)** high SDI regions. Abbreviations: MDR, multidrug-resistant; TB, tuberculosis; SDI, socio-demographic index.

Globally in 2019, the ASR of MDR-TB was 5.63 (95% UI = 3.12–9.73) per 100,000 among 450,600 cases (95% UI = 247,830–785,370), and the MDR incidence rate was 5.30% (Table 1). The distribution of cases during 2010–2019 was almost U-shaped (Figure 1A). The ASR decreased on average by 1.36% (EAPC = −1.36, 95% CI = −2.19 to −0.52) per year during 2010–2019 (Table 1).

**TABLE 1:** The cases and ASR for incidence of MDR-TB and XDR-TB in 2019, their temporal incident trends from 2010 to 2019, and MDR and XDR rate of TB.

Characteristics	MDR-TB				XDR-TB			
	Incident cases	ASR per 100,000	EAPC	MDR rate	Incident cases	ASR per 100,000	EAPC	XDR rate
	No. × 10^3^ (95% UI)	No. (95% UI)	No. (95% CI)	%	No. × 10^3^ (95% UI)	No. (95% UI)	No. (95% CI)	%
Global	450.6 (247.83–785.37)	5.63 (3.12–9.73)	−1.36 (−2.19–−0.52)	5.30	25.06 (17.09–36.47)	0.31 (0.21–0.45)	0.69 (−0.15–1.54)	0.29
Socio-demographic index								
Low	83.78 (48.55–144.44)	9.53 (5.27–16.86)	0.07 (−0.19–0.34)	4.15	1.36 (0.64–2.69)	0.16 (0.07–0.33)	4.44 (4.27–4.61)	0.07
Low-middle	173.14 (67.91–377.69)	10.32 (4.01–22.66)	−0.26 (−0.53–0.02)	5.47	5.24 (2.44–10.26)	0.31 (0.15–0.62)	3.8 (3.72–3.89)	0.17
Middle	111.4 (55.2–207.28)	4.43 (2.22–8.16)	−2.18 (−3.51–−0.82)	4.58	6.16 (3.6–10.33)	0.24 (0.15–0.41)	1.57 (0.43–2.73)	0.25
Middle-high	79.55 (47.63–125.98)	4.83 (2.89–7.73)	−4.72 (−5.93–−3.5)	10.50	12.01 (8–17.15)	0.72 (0.48–1.02)	−0.69 (−1.64–0.27)	1.58
High	2.64 (1.6–4.56)	0.22 (0.14–0.39)	−2.83 (−3.55–−2.11)	2.13	0.29 (0.19–0.48)	0.02 (0.02–0.04)	1.34 (0.84–1.85)	0.24
Region								
Asia Pacific–high income	0.58 (0.14–1.84)	0.2 (0.05–0.64)	−3.62 (−4.13–−3.12)	1.22	0.07 (0.02–0.23)	0.03 (0.01–0.08)	1.26 (0.88–1.64)	0.15
Central Asia	10.68 (7.03–14.94)	11.42 (7.55–16.05)	−5.86 (−6.23–−5.49)	20.51	2.34 (1.54–3.27)	2.5 (1.65–3.52)	−0.56 (−0.74–−0.38)	4.49
East Asia	31.26 (7.07–95.81)	1.81 (0.41–5.52)	−6.8 (−9.18–−4.36)	4.10	2.85 (0.64–8.74)	0.16 (0.04–0.5)	−2.24 (−4.49–0.05)	0.37
South Asia	257.75 (72.44–595.41)	14.87 (4.18–33.94)	−0.39 (−0.84–0.05)	6.76	6.32 (1.78–14.59)	0.36 (0.1–0.83)	4.29 (4.04–4.54)	0.17
Southeast Asia	25.34 (14.88–41.38)	3.78 (2.23–6.16)	-1.25 (-2.23–0.27)	2.28	2.31 (1.36–3.77)	0.35 (0.2–0.56)	3.73 (2.91–4.56)	0.21
Australasia	0.05 (0.02–0.1)	0.17 (0.07–0.34)	4.25 (2.69–5.82)	2.75	0.01 (0–0.01)	0.02 (0.01–0.04)	9.44 (7.53–11.39)	0.35
Caribbean	0.09 (0.03–0.23)	0.18 (0.06–0.48)	4.71 (3.03–6.42)	0.56	0.01 (0–0.02)	0.01 (0.01–0.04)	9.92 (7.86–12.03)	0.04
Central Europe	0.41 (0.22–0.72)	0.29 (0.16–0.52)	−4.41 (−5.27–−3.55)	2.03	0.09 (0.05–0.16)	0.06 (0.03–0.11)	0.73 (-0.43–1.9)	0.44
Eastern Europe	44.87 (28.33–65.58)	18.87 (11.81–27.74)	−4.8 (−5.78–−3.81)	27.18	9.83 (6.21–14.37)	4.13 (2.59–6.08)	0.55 (−0.23–1.34)	5.96
Western Europe	0.68 (0.46–1)	0.16 (0.11–0.23)	−2.19 (−2.28–−2.1)	2.26	0.09 (0.06–0.13)	0.02 (0.01–0.03)	2.83 (2.62–3.05)	0.28
Andean Latin America	2.71 (1.5–4.57)	4.26 (2.36–7.14)	−4.09 (−6.16–−1.97)	6.46	0.21 (0.12–0.36)	0.34 (0.19–0.56)	0.56 (−1.34–2.49)	0.51
Central Latin America	1.35 (0.57–2.68)	0.54 (0.23–1.06)	0.76 (0.37–1.15)	2.97	0.11 (0.05–0.21)	0.04 (0.02–0.08)	5.75 (5.41–6.09)	0.23
Southern Latin America	0.11 (0.03–0.35)	0.16 (0.04–0.51)	−1.68 (−2.04–−1.33)	1.34	0.01 (0–0.04)	0.02 (0.01–0.06)	3.33 (3.05–3.62)	0.17
Tropical Latin America	2.05 (0.44–5.58)	0.86 (0.18–2.34)	3.22 (2.78–3.66)	3.07	0.16 (0.03–0.44)	0.07 (0.01–0.18)	8.36 (7.77–8.96)	0.24
North Africa and Middle East	4.45 (2.69–7.56)	0.76 (0.46–1.27)	−2.88 (−3.24–−2.51)	2.90	0.16 (0.09–0.27)	0.03 (0.02–0.04)	1.84 (1.69–2)	0.10
North America–high income	0.13 (0.06–0.27)	0.03 (0.01–0.07)	−3.3 (−4.37–−2.22)	1.36	0.02 (0.01–0.03)	0 (0–0.01)	1.71 (0.79–2.63)	0.17
Oceania	0.42 (0.16–0.97)	3.53 (1.32–7.97)	11.09 (5.12–17.4)	3.43	0.04 (0.01–0.09)	0.32 (0.12–0.73)	16.14 (9.49–23.19)	0.31
Central Sub-Saharan Africa	7.84 (2.09–22.46)	7.38 (1.96–21.23)	1.16 (0.72–1.6)	2.42	0.05 (0.01–0.14)	0.05 (0.01–0.14)	5.8 (5.1–6.5)	0.02
Eastern Sub-Saharan Africa	29.93 (18.85–50.47)	9.17 (5.76–15.59)	1.9 (1.6–2.2)	3.29	0.19 (0.12–0.33)	0.06 (0.04–0.1)	6.59 (6.05–7.13)	0.02
Southern Sub-Saharan Africa	9.69 (4.84–18.68)	11.54 (5.82–22.19)	−0.83 (−2.35–0.72)	3.35	0.06 (0.03–0.12)	0.07 (0.04–0.14)	3.77 (2.41–5.15)	0.02
Western Sub-Saharan Africa	20.2 (9.45–40.6)	5.95 (2.77–12.22)	−2.7 (−3.28–−2.12)	3.29	0.13 (0.06–0.26)	0.04 (0.02–0.08)	1.89 (1.49–2.31)	0.02

Abbreviations: MDR, multidrug-resistant; XDR, extensively drug-resistant; TB, tuberculosis; ASR, age-standardized rate; CI, confidence interval; EAPC, estimated annual percentage change; UI, uncertainty interval.

For SDI regions, the ASR exhibited a stable trend in the low and low-middle SDI regions and decreased in the other three SDI regions (Table 1). The high SDI regions had the fewest cases, and the lowest ASR and MDR rates (Table 1; Figure 1). The number of MDR-TB cases increased monotonically in the low- and low-middle SDI regions (Figure 1).

The ASR of MDR-TB in 12 of the 21 geographical regions exhibited decreasing trends during 2010–2019, with the largest decrease observed in East Asia (EAPC = −6.8, 95% CI = −9.18 to −4.36), followed by Central Asia and Eastern Europe (Table 1). However, there were still two regions with stable ASR, and even seven with increased ASR (Table 1). Oceania had the largest increase (EAPC = 11.09, 95% CI = 5.12–17.4). Central Asia and Eastern Europe had the highest MDR rates, at 20.51% and 27.18%, respectively (Table 1).

The incidence trend of MDR-TB varied among the 204 countries and territories, decreasing in 116 of them, remaining stable in 35, and increasing in 53 (Supplementary Table S1). Countries with high MDR rates were mostly in Eastern Europe and Central Asia, which was consistent with the analysis at the regional level. For example, the ten regions with the highest MDR rates (in decreasing order) were the Republic of Moldova (MDR rate = 37.87%), Belarus (36.91%), Ukraine (29.52%), Russian Federation (26.14%), Kyrgyzstan (25.46%), Uzbekistan (23.92%), Kazakhstan (20.60%), Azerbaijan (20.35%), Georgia (18.12%), and Estonia (17.58%) (Supplementary Table S1).

### Extensively drug-resistant tuberculosis

**FIGURE 2:**
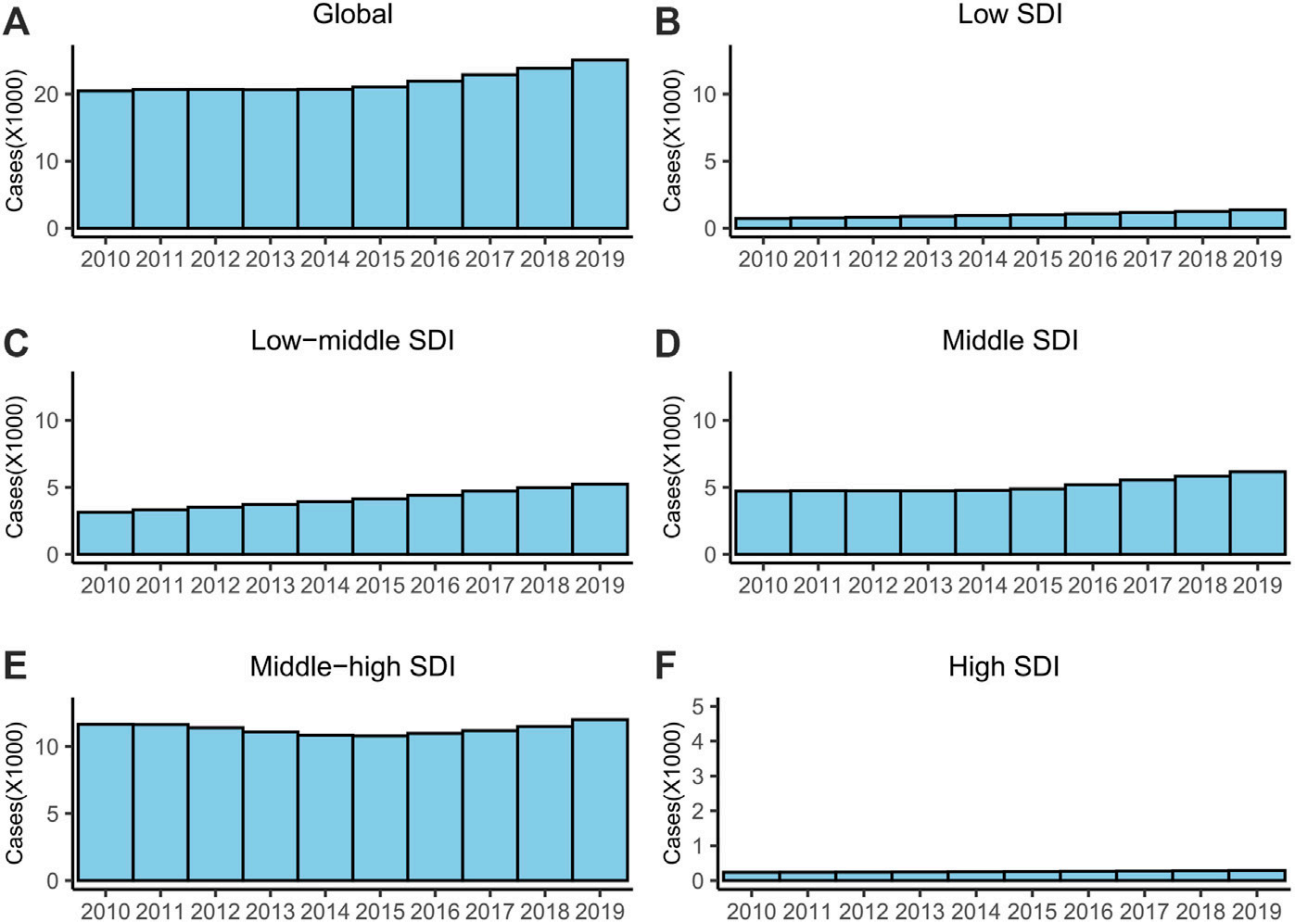
The cases of XDR-TB from 2010 to 2019. **(A)** Global, **(B)** low SDI regions, **(C)** low-middle SDI regions, **(D)** middle SDI regions, **(E)** middle-high SDI regions and **(F)** high SDI regions. Abbreviations: XDR, extensively drug-resistant; TB, tuberculosis; SDI, socio-demographic index.

In 2019, there were 25,060 (95% UI = 17,090–36,470) new XDR-TB cases globally, which had increased by 22.5% compared with 2010, and the ASR was 0.31 (95% UI = 0.21–0.45) per 100,000 (Table 1; Figure 2A). The XDR rate was 0.29%. The ASR was stable during 2010–2019 (EAPC = 0.69, 95% CI = −0.15–1.54) (Table 1).

For SDI regions, the ASR was only stable in the middle-high SDI regions (Table 1). The ASRs and numbers of cases increased in the other four SDI regions (Table 1; Figure 2). The middle-high SDI had the most XDR-TB cases, and the highest ASR and XDR rates (Table 1; Figure 2). The high SDI region had the fewest XDR-TB cases and lowest ASR rate (Table 1; Figure 2).

The ASRs increased in 16 of the 21 geographical regions, was stable in 4, and decreased only in Central Asia. The increase was largest in Oceania (EAPC = 16.14, 95% CI = 9.49–23.19), followed by the Caribbean (EAPC = 9.92, 95% CI = 7.86–12.03) and Australasia (EAPC = 9.44, 95% CI = 7.53–11.39) (Table 1). Although the ASR decreased in Central Asia, its XDR rate was the second highest (XDR rate = 4.49%), and that of Eastern Europe was the highest (XDR rate = 5.96%).

The trend of ASR varied among the 204 countries and territories. The ASRs of most countries and territories (144 of 204) increased, while those of 24 were stable and it decreased in 36 (Supplementary Table S1). There was a close correspondence between countries with high MDR rates and high XDR rates; for example, the ten countries with the highest XDR rates also had the ten highest MDR rates (Supplementary Table S1).

### Age distributions of MDR-TB and XDR-TB incidence rates, and MDR and XDR rates

**FIGURE 3:**
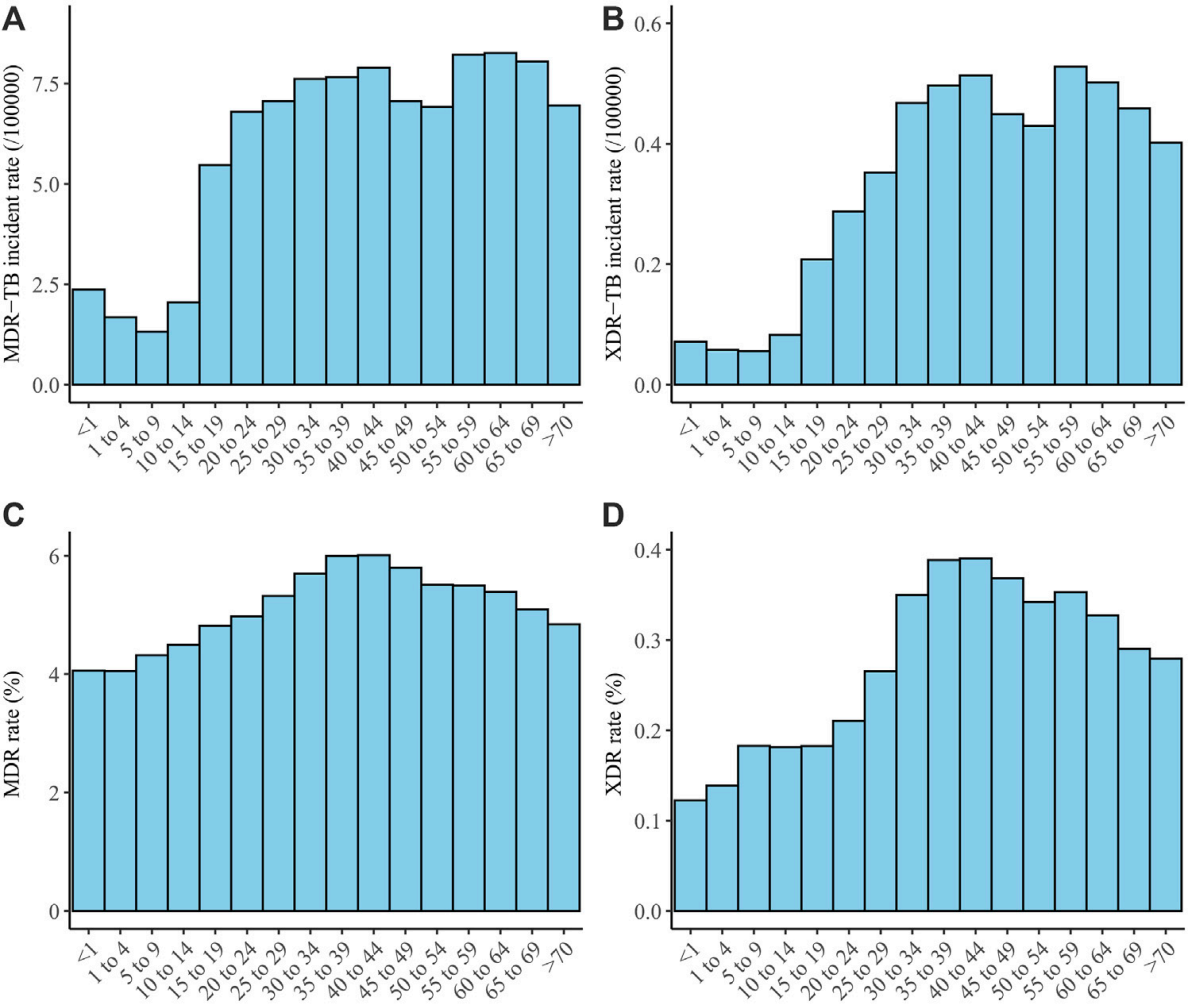
Age distribution of MDR-TB incidence rate **(A)**, XDR-TB incidence rate **(B)**, MDR rate **(C)**, and XDR rate **(D)**. Abbreviations: MDR, multidrug-resistant; TB, tuberculosis; XDR, extensively drug-resistant.

The age distributions of the MDR and XDR incidence rates were similar, with both having two peaks. The MDR-TB incidence rate peaked in those aged 35–44 and 55–64 years. The XDR-TB incidence rates were similar, also peaking in those aged 35–44 and 55–64 years (Figures 3A, B). The MDR and XDR rates both peaked in those aged 35–44 years (Figures 3C, D).

### Relationships among the ASRs of MDR-TB and XDR-TB, MDR and XDR rates, and SDI

**FIGURE 4:**
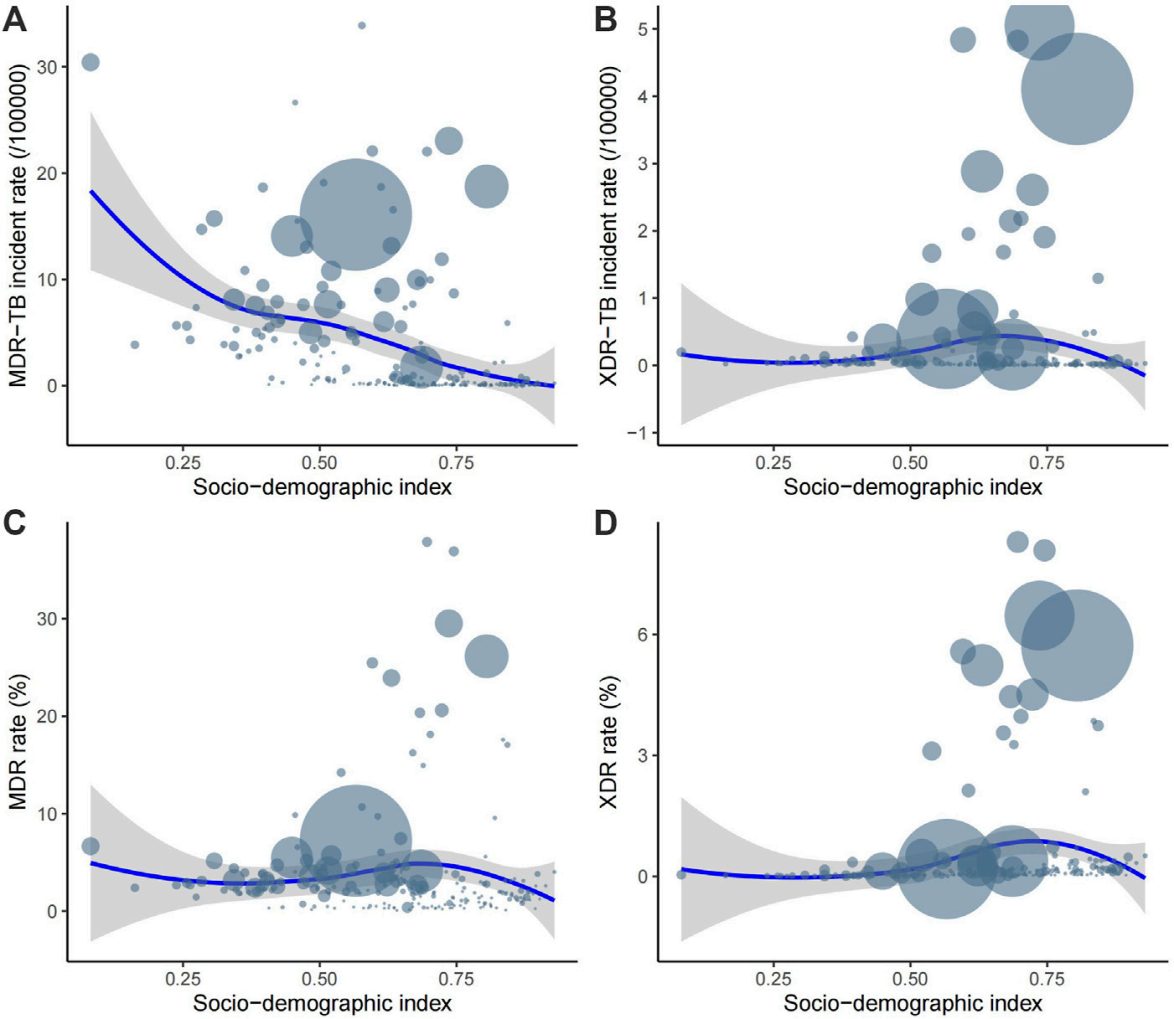
The correlation between ASR of MDR-TB **(A)**, ASR of XDR-TB **(B)**, MDR rate **(C)**, and XDR rate **(D)** and SDI. Each circle represents a country or territory. The size of the circle represents the number of cases. Abbreviations: ASR, age-standardized rate; MDR, multidrug-resistant; TB, tuberculosis; XDR, extensively drug-resistant; SDI, socio-demographic index.

We analyzed the relationships among the ASRs of MDR-TB and XDR-TB, MDR and XDR rates, and SDI based on national-level data. A significant negative correlation was found between the ASR of MDR-TB and SDI (*p* < 0.001, *ρ* = −0.43) (Figure 4A). No significant relationship was found between the ASR of XDR-TB (*p* = 0.54, *ρ* = 0.04) or the MDR incidence rate (*p* = 0.86, *ρ* = 0.01) and SDI (Figures 4B, C). A significant positive correlation was found overall between the XDR rate and SDI (*p* = 0.03, *ρ* = 0.15) (Figure 4D). However, as shown in Figure 4D, there was a negative relationship between them when the SDI exceeded about 0.75.

## Discussion

MDR-TB and XDR-TB are serious problems that represent great threats and challenges to human and public health (Akkerman et al., 2019; Borisov et al., 2019; Shang et al., 2022). According to the End TB Strategy, TB incidence and mortality should have declined by at least 20% and 35%, respectively, between 2015 and 2020. However, the performance of the strategy has been suboptimal, with only 11% and 9.2% declines in TB incidence and mortality, respectively, by 2021 (Jeremiah et al., 2022). MDR-TB and XDR-TB played important roles in this poor performance (Seung et al., 2015; Jeremiah et al., 2022). In the present study, we analyzed the global incidence trends of MDR-TB and XDR-TB during 1990–2019 to help improve the current status of TB based on the GBD 2019 database.

The analyzed GBD database contains data from 1990 to 2019. We were more concerned about the current trend than the previous trend, and so this study focused on the data from the last 10-year period covered in the GBD. Previous data may affect the actual recent trends. For example, if the EAPC during 1990–2009 was significantly negative and that during 2010–2019 was significantly positive, it is possible that the EAPC during 1990–2019 would be significantly negative. Although this study found that the ASR of MDR-TB worldwide is declining, the current annual reduction in global TB incidence is 2%, which is too slow to achieve an end to the epidemic in the foreseeable future. According to the End TB Strategy (Uplekar et al., 2015; World Health Organization, 2019), the annual decline in global TB incidence rates must increase to 10% annually by 2025. However, the EAPC of MDR-TB was −1.36, meaning that the ASR of MDR-TB decreased by 1.36% per year, which is far less than 10% or even 2%. This study found that the high ASR of MDR-TB was mostly attributable to Eastern Europe, South Asia, Southern Sub-Saharan Africa, Central Asia, Eastern Sub-Saharan Africa, and Central Sub-Saharan Africa. There are several possible reasons for the higher ASR of MDR-TB in these regions. The economic level is low and the accessibility to public health services is poor in these regions, and it includes many developing countries, which may have problems such as poverty, malnutrition, and poor living conditions (Lange et al., 2018). Our results also indicated that the ASR of MDR-TB had a significant negative correlation with SDI. SDI is a composite measure of income *per capita*, total fertility rate (age <25 years), and average education level (for those aged ≥15 years), and is used as a measure of sociodemographic development (GBD 2019 Risk Factors Collaborators, 2020; GBD 2019 Diabetes in the Americas Collaborators, 2022). As is well known, the incidence rate of TB is related to the socioeconomic and development levels (Nordholm et al., 2022; Soares et al., 2022). This affects the incidence rate of TB in various aspects. For example, public health systems are imperfect and the prevention and control of infectious diseases is weak in regions with low SDI. Both in terms of resource allocation and professional talent training, there are problems such as insufficient quantity, low quality, and unreasonable structure (Liu et al., 2019), which lead to an increase in the incidence rate of TB that can cause an increase in the incidence rate of MDR-TB. According to our analysis, the impact of social development level on the incidence rate of MDR-TB may be mostly attributed to its impact on the incidence rate of TB, rather than directly on that of MDR. We did not observe a significant correlation between MDR rate and SDI in this study. Eastern Europe and Central Asia had the highest MDR rates, which was consistent with previous reports (Dirlikov et al., 2015; Lange et al., 2018). It is promising that these regions had larger downward trends for the ASR of their MDR-TB compared with most regions. According to our results, XDR-TB should be considered because its ASR had no downward trend and actually increased in most regions. The trend was only declining in Central Asia. Regions with high MDR rate tend to have a high XDR rate, which was consistent with the principle of drug resistance in biology. There are two types of drug resistance in *Mycobacterium tuberculosis*: genetic and phenotypic resistance. Genetic drug resistance is caused by mutations in chromosomal genes in bacterial growth, while phenotypic resistance or drug tolerance is caused by epigenetic changes in gene expression and protein modification that induce drug tolerance in non-growing bacterial persisters (Zhang and Yew, 2015). These two types are mostly caused by drug use. A high MDR rate may result in a high XDR rate by increasing the use of second-line drugs. In the present study, XDR-TB and MDR-TB had different relationships with SDI; that is, the XDR rate had a significant positive correlation with SDI, but the ASR of XDR-TB was not significantly correlated with SDI. Although the relationship between XDR rate and SDI was significant, it was not strong, with a *ρ* value of only 0.15. The *ρ* value represents the strength of the correlation in Pearson’s coefficient (Pearson, 1920; Rodgers and Nicewander, 1988). This may mean that the XDR rate was more affected by other factors. We also analyzed the age distribution of MDR-TB and XDR-TB. The results indicated that there were two peaks for the incidence rates of MDR-TB and XDR-TB, in those aged 35–44 and 55–64 years. Nevertheless, there were also peaks for MDR and XDR rates, in those aged 35–44 years. The peaks for MDR and XDR rates were consistent with the first peaks of the incidence rates of MDR-TB and XDR-TB, which was logical since a high drug resistance leads to a high incidence rate in drug-resistant TB. A reasonable explanation for the absence of second peaks for MDR and XDR rates is that the mortality rate of TB is high among the elderly (Dhamnetiya et al., 2021), resulting in a small proportion of elderly patients having received previous treatment for TB. Drug-resistant TB mostly occurs in patients previously treated for TB (World Health Organization, 2021a). At the national level, India, China, and the Russian Federation are the countries with the three largest numbers of MDR-TB and XDR-TB cases, which account for most new cases in the world. This result for MDR-TB was consistent with a WHO report (World Health Organization, 2021b). WHO do not report the global incidence of XDR-TB, which is rarely reported. The incidence trends of MDR-TB and XDR-TB in China were declining. The incidence trend of MDR-TB in the Russian Federation was declining, while that of XDR was stable. India should receive more attention, because it has the most MDR-TB cases with a stable incidence trend and the second-highest rate of XDR-TB cases with an increasing incidence trend. Improving the incidence trends of MDR-TB and XDR-TB in India is important to improve control of the global incidence rates of MDR-TB and XDR-TB.

This study had several limitations, most notably being that the participants were from the GBD database and calculations were made using a model based on existing data in each country; that is, where data were not available, the results depended on predictive validity of the model for out-of-sample data. In addition, the MDR or XDR rate was the ratio of new MDR- or XDR-TB cases to new TB cases. Cases were point estimates, and their 95% UIs were determined through 1,000 calculations. This approach made it impossible to estimate the UIs or CIs of MDR and XDR rates.

The present study has performed the most comprehensive analysis of the global trends of MDR-TB and XDR-TB during 2010–2019. Although the incidence of MDR-TB was declining, the rate of decline was too slow; moreover, the incidence trend of XDR-TB was not declining. The incidence trends of MDR-TB and XDR-TB varied markedly among different regions and countries. High-risk age groups, regions and countries with high burdens, and low-SDI regions require careful consideration, and effective tools need to be developed to curb MDR-TB and XDR-TB.

## Data Availability

The original contributions presented in the study are included in the article/Supplementary Material, further inquiries can be directed to the corresponding author.
