# Supplementary material for: Global trends in the incidence rates of MDR and XDR tuberculosis: Findings from the global burden of disease study 2019
Source: Front Pharmacol. 2023 Feb 24;14:1156249. doi: 10.3389/fphar.2023.1156249 (PMC9998482; doi:10.3389/fphar.2023.1156249)
Supplement: Supplementary file 1 [file Table1.DOCX]

**Table S1** The cases and ASR for incidence of MDR-TB and XDR-TB in 2019, their temporal incident trends from 2010 to 2019, and MDR and XDR rate of TB at country and territory level.

|  | **MDR-TB** | | | |  | **XDR-TB** | | | |
| --- | --- | --- | --- | --- | --- | --- | --- | --- | --- |
|  | **Incident cases** | **ASR per 100,000** | **EAPC** | **MDR rate** |  | **Incident cases** | **ASR per 100,000** | **EAPC** | **XDR rate** |
|  | **No.(95% UI)** | **No.(95% UI)** | **No.(95% CI)** | **%** |  | **No.(95% UI)** | **No.(95% UI)** | **No.(95% CI)** | **%** |
| Afghanistan | 1042.19(180.67–3419.32) | 3.74(0.64–12.4) | -5.83(-7.03–-4.62) | 4.39 |  | 36.78(6.38–120.69) | 0.13(0.02–0.44) | -1.12(-2.13–-0.1) | 0.16 |
| Albania | 0.73(0.14–2.12) | 0.02(0–0.06) | -11.17(-13.01–-9.29) | 0.76 |  | 0.16(0.03–0.46) | 0(0–0.01) | -6.11(-7.83–-4.37) | 0.17 |
| Algeria | 140.28(12.81–663.16) | 0.35(0.03–1.64) | 1.07(-0.02–2.17) | 2.03 |  | 4.95(0.45–23.4) | 0.01(0–0.06) | 5.79(4.35–7.26) | 0.07 |
| American Samoa | 0.02(0–0.1) | 0.04(0–0.18) | -0.67(-1.47–0.13) | 0.30 |  | 0(0–0.01) | 0(0–0.02) | 4.35(3.47–5.24) | 0.03 |
| Andorra | 0.01(0–0.03) | 0.01(0–0.03) | -6.23(-7.73–-4.7) | 0.25 |  | 0(0–0) | 0(0–0) | -1.46(-2.83–-0.08) | 0.03 |
| Angola | 1806.57(236.96–6617.75) | 7.62(0.98–26.97) | -1.7(-1.8–-1.6) | 2.87 |  | 11.66(1.53–42.7) | 0.05(0.01–0.17) | 2.85(2.53–3.17) | 0.02 |
| Antigua and Barbuda | 0.13(0.01–0.58) | 0.13(0.01–0.61) | -0.68(-1.86–0.51) | 0.71 |  | 0.01(0–0.05) | 0.01(0–0.05) | 4.21(2.63–5.81) | 0.06 |
| Argentina | 86.86(12.29–318.17) | 0.19(0.03–0.69) | -1.82(-2.18–-1.45) | 1.55 |  | 10.92(1.55–40.02) | 0.02(0–0.09) | 3.22(2.81–3.63) | 0.20 |
| Armenia | 116.23(24.66–272.13) | 3.47(0.72–8.19) | -8.19(-9.81–-6.54) | 14.94 |  | 25.47(5.4–59.62) | 0.76(0.16–1.79) | -2.83(-4.28–-1.34) | 3.27 |
| Australia | 42.77(17.84–90.79) | 0.16(0.07–0.35) | 5.42(4.01–6.85) | 3.11 |  | 5.38(2.24–11.41) | 0.02(0.01–0.04) | 10.73(8.99–12.49) | 0.39 |
| Austria | 16.59(6.27–35.18) | 0.19(0.07–0.4) | -5.96(-6.51–-5.41) | 3.12 |  | 2.09(0.79–4.42) | 0.02(0.01–0.05) | -0.92(-1.5–-0.33) | 0.39 |
| Azerbaijan | 1096.27(343.45–2105.36) | 9.79(3.05–18.83) | 0.42(-0.3–1.15) | 20.35 |  | 240.2(75.21–461.35) | 2.14(0.67–4.13) | 5.8(4.82–6.79) | 4.46 |
| Bahamas | 0.73(0.1–2.73) | 0.18(0.03–0.69) | 2.89(-0.74–6.66) | 1.54 |  | 0.06(0.01–0.22) | 0.01(0–0.05) | 7.58(3.41–11.91) | 0.12 |
| Bahrain | 6.93(1.13–20.95) | 0.48(0.08–1.43) | -2.26(-5.02–0.59) | 2.85 |  | 0.24(0.04–0.74) | 0.02(0–0.05) | 2.35(-0.85–5.65) | 0.10 |
| Bangladesh | 7779.63(1870.31–19146.36) | 5.02(1.21–12.38) | -1.27(-1.73–-0.8) | 3.60 |  | 190.68(45.87–469.31) | 0.12(0.03–0.3) | 3.55(3.23–3.88) | 0.09 |
| Barbados | 0.07(0.01–0.34) | 0.02(0–0.11) | 1.67(0.32–3.05) | 0.34 |  | 0.01(0–0.03) | 0(0–0.01) | 6.72(4.93–8.55) | 0.03 |
| Belarus | 987.76(661.19–1366.44) | 8.69(5.93–12.09) | -5.36(-6.12–-4.6) | 36.91 |  | 216.43(144.88–299.49) | 1.9(1.3–2.65) | 0.04(-0.49–0.57) | 8.09 |
| Belgium | 14.77(4.84–35.76) | 0.13(0.04–0.32) | -3.95(-4.67–-3.22) | 1.79 |  | 1.86(0.61–4.5) | 0.02(0.01–0.04) | 1.1(0.57–1.63) | 0.22 |
| Belize | 0.47(0.06–2.04) | 0.12(0.01–0.54) | 3.32(1.21–5.48) | 0.50 |  | 0.04(0–0.16) | 0.01(0–0.04) | 8.45(5.87–11.09) | 0.04 |
| Benin | 259.96(47.92–726.66) | 2.76(0.5–7.65) | 0.61(0.21–1.01) | 1.87 |  | 1.68(0.31–4.69) | 0.02(0–0.05) | 5.47(5.05–5.9) | 0.01 |
| Bermuda | 0.06(0–0.39) | 0.09(0.01–0.52) | 5.32(2.23–8.5) | 0.53 |  | 0.01(0–0.03) | 0.01(0–0.04) | 10.37(6.67–14.19) | 0.04 |
| Bhutan | 29.29(4.15–95.74) | 4.01(0.57–13.18) | -3.21(-3.38–-3.04) | 4.04 |  | 0.72(0.1–2.35) | 0.1(0.01–0.32) | 1.34(0.94–1.74) | 0.10 |
| Bolivia (Plurinational State of) | 466.35(68.86–1513.57) | 4.14(0.62–13.33) | 1.63(-0.38–3.67) | 4.74 |  | 36.83(5.44–119.55) | 0.33(0.05–1.05) | 6.62(4.13–9.18) | 0.37 |
| Bosnia and Herzegovina | 2.58(0.69–6.97) | 0.06(0.01–0.15) | -5.6(-6.68–-4.51) | 0.46 |  | 0.57(0.15–1.53) | 0.01(0–0.03) | -0.45(-1.36–0.47) | 0.10 |
| Botswana | 421.58(78.78–1323.72) | 16.55(3.1–50.11) | 2.89(2.35–3.45) | 4.80 |  | 2.72(0.51–8.53) | 0.11(0.02–0.32) | 7.65(6.85–8.46) | 0.03 |
| Brazil | 1981.59(371.4–5480.54) | 0.85(0.16–2.37) | 3.23(2.8–3.68) | 3.07 |  | 156.5(29.33–432.89) | 0.07(0.01–0.19) | 8.38(7.79–8.97) | 0.24 |
| Brunei Darussalam | 0.64(0.1–2.08) | 0.16(0.03–0.54) | 4.61(0.58–8.8) | 0.33 |  | 0.08(0.01–0.26) | 0.02(0–0.07) | 9.84(5.24–14.63) | 0.04 |
| Bulgaria | 28.13(7.66–67.62) | 0.34(0.09–0.79) | -11.17(-12.22–-10.11) | 2.38 |  | 6.16(1.68–14.82) | 0.07(0.02–0.17) | -5.91(-6.62–-5.18) | 0.52 |
| Burkina Faso | 975.24(143.13–3215.72) | 5.64(0.84–18.66) | -2.83(-3.68–-1.98) | 2.90 |  | 6.29(0.92–20.74) | 0.04(0.01–0.12) | 1.82(1.18–2.45) | 0.02 |
| Burundi | 1298.82(180.33–4382.93) | 14.71(2.1–50.51) | 2.16(1.62–2.71) | 3.06 |  | 8.38(1.16–28.28) | 0.09(0.01–0.33) | 6.84(6.06–7.63) | 0.02 |
| Cabo Verde | 16.62(2.29–51.21) | 3.12(0.43–9.48) | -2.99(-3.91–-2.06) | 3.33 |  | 0.11(0.01–0.33) | 0.02(0–0.06) | 1.43(0.73–2.14) | 0.02 |
| Cambodia | 337.85(50.49–1120.04) | 2.23(0.33–7.43) | -9.32(-12.78–-5.72) | 0.72 |  | 30.81(4.6–102.1) | 0.2(0.03–0.68) | -4.93(-8.35–-1.38) | 0.07 |
| Cameroon | 800.03(114.36–2472.29) | 3.52(0.51–10.68) | -3.44(-4.18–-2.69) | 2.28 |  | 5.16(0.74–15.96) | 0.02(0–0.07) | 1.16(0.6–1.72) | 0.01 |
| Canada | 21.94(7.86–49.54) | 0.05(0.02–0.12) | -0.51(-1.05–0.04) | 1.23 |  | 2.76(0.99–6.23) | 0.01(0–0.02) | 4.54(4.11–4.98) | 0.15 |
| Central African Republic | 328.64(52.17–1084.8) | 7.36(1.15–24.32) | 0.42(0.08–0.75) | 1.45 |  | 2.12(0.34–7) | 0.05(0.01–0.16) | 5.03(4.45–5.62) | 0.01 |
| Chad | 678(93.91–2057.93) | 5.67(0.78–17.41) | -2.98(-3.28–-2.68) | 2.67 |  | 4.37(0.61–13.27) | 0.04(0.01–0.11) | 1.62(1.47–1.77) | 0.02 |
| Chile | 22.39(7.44–52.4) | 0.11(0.04–0.26) | -2.08(-3.27–-0.87) | 1.08 |  | 2.81(0.94–6.59) | 0.01(0–0.03) | 2.84(1.83–3.86) | 0.14 |
| China | 29612.85(5484.88–94639.5) | 1.77(0.33–5.63) | -7.04(-9.48–-4.54) | 4.12 |  | 2700.14(500.12–8629.62) | 0.16(0.03–0.51) | -2.5(-4.81–-0.14) | 0.38 |
| Colombia | 406.33(68.57–1096.7) | 0.79(0.13–2.14) | 0.63(-0.13–1.4) | 4.26 |  | 32.09(5.42–86.64) | 0.06(0.01–0.17) | 5.5(4.47–6.55) | 0.34 |
| Comoros | 170.41(21.46–557.46) | 26.63(3.42–86.97) | 13.16(10.31–16.08) | 9.86 |  | 1.1(0.14–3.6) | 0.17(0.02–0.56) | 18.11(14.99–21.31) | 0.06 |
| Congo | 213.45(25.06–764.92) | 4.74(0.56–16.59) | 1.85(1.2–2.5) | 2.03 |  | 1.38(0.16–4.94) | 0.03(0–0.11) | 6.48(5.58–7.39) | 0.01 |
| Cook Islands | 0.01(0–0.04) | 0.05(0–0.22) | 0.2(-0.2–0.61) | 0.28 |  | 0(0–0) | 0(0–0.02) | 5.33(4.65–6.02) | 0.03 |
| Costa Rica | 7.58(1.07–26.38) | 0.15(0.02–0.52) | -3.73(-5.39–-2.04) | 1.96 |  | 0.6(0.08–2.08) | 0.01(0–0.04) | 0.93(-0.58–2.47) | 0.15 |
| Croatia | 1.49(0.34–4.23) | 0.03(0.01–0.07) | -11.25(-14.32–-8.07) | 0.36 |  | 0.33(0.08–0.93) | 0.01(0–0.02) | -6.53(-9.59–-3.36) | 0.08 |
| Cuba | 15.61(3.1–44.39) | 0.12(0.02–0.34) | 2.04(1.76–2.32) | 2.33 |  | 1.23(0.24–3.51) | 0.01(0–0.03) | 7.02(6.91–7.12) | 0.18 |
| Cyprus | 0.76(0.15–2.26) | 0.05(0.01–0.16) | -17.39(-19.8–-14.9) | 1.90 |  | 0.1(0.02–0.28) | 0.01(0–0.02) | -12.92(-15.21–-10.56) | 0.24 |
| Czechia | 10.57(3.76–24.32) | 0.08(0.03–0.18) | -5.12(-6.29–-3.94) | 1.98 |  | 2.32(0.82–5.33) | 0.02(0.01–0.04) | 0.15(-0.86–1.17) | 0.43 |
| Côte d'Ivoire | 1138.15(177.24–3895.63) | 5.48(0.82–18.15) | -0.88(-1.49–-0.27) | 3.22 |  | 7.34(1.14–25.13) | 0.04(0.01–0.12) | 3.68(3.17–4.19) | 0.02 |
| Democratic People's Republic of Korea | 1399.99(449.71–3193.85) | 4.84(1.55–11.01) | -1.22(-2.06–-0.38) | 4.32 |  | 127.65(41.02–291.24) | 0.44(0.14–1) | 3.56(2.93–4.19) | 0.39 |
| Democratic Republic of the Congo | 5332.97(668.32–19684.56) | 7.54(0.95–27.42) | 2.26(1.65–2.87) | 2.40 |  | 34.41(4.31–127) | 0.05(0.01–0.18) | 6.93(6.06–7.81) | 0.02 |
| Denmark | 4.98(1.47–12.81) | 0.08(0.02–0.22) | 0.51(-0.77–1.8) | 1.78 |  | 0.63(0.19–1.61) | 0.01(0–0.03) | 5.44(3.8–7.11) | 0.22 |
| Djibouti | 159.12(67.58–321.56) | 15.49(6.57–31.46) | 1.91(0.79–3.04) | 6.56 |  | 1.03(0.44–2.07) | 0.1(0.04–0.2) | 6.53(5.11–7.97) | 0.04 |
| Dominica | 0.12(0.01–0.52) | 0.16(0.01–0.71) | 3.05(1.32–4.81) | 0.68 |  | 0.01(0–0.04) | 0.01(0–0.06) | 8.28(6.14–10.46) | 0.05 |
| Dominican Republic | 24.47(2.48–116.2) | 0.22(0.02–1.07) | 7.41(4.51–10.39) | 0.53 |  | 1.93(0.2–9.18) | 0.02(0–0.08) | 12.81(9.43–16.3) | 0.04 |
| Ecuador | 307.76(56.98–930.79) | 1.76(0.33–5.39) | -3.55(-4.8–-2.29) | 5.06 |  | 24.31(4.5–73.49) | 0.14(0.03–0.43) | 1.16(0.08–2.24) | 0.40 |
| Egypt | 567.97(127.85–1475.94) | 0.62(0.14–1.58) | -4.34(-5.06–-3.62) | 4.56 |  | 20.05(4.51–52.12) | 0.02(0–0.06) | 0.42(-0.07–0.91) | 0.16 |
| El Salvador | 13.79(1.73–53.29) | 0.22(0.03–0.85) | -2.48(-2.93–-2.04) | 1.82 |  | 1.09(0.14–4.21) | 0.02(0–0.07) | 2.33(2.08–2.58) | 0.14 |
| Equatorial Guinea | 46.44(5.85–145.66) | 4.04(0.51–12.98) | 1.91(1.53–2.28) | 2.53 |  | 0.3(0.04–0.94) | 0.03(0–0.08) | 6.55(5.93–7.17) | 0.02 |
| Eritrea | 969.96(141.44–3210.3) | 18.65(2.78–62) | 0.83(-0.15–1.83) | 3.83 |  | 6.26(0.91–20.71) | 0.12(0.02–0.4) | 5.39(4.09–6.71) | 0.02 |
| Estonia | 34.08(17.58–57.48) | 2.23(1.15–3.78) | -9.99(-11.87–-8.08) | 17.58 |  | 7.47(3.85–12.59) | 0.49(0.25–0.83) | -4.75(-6.47–-3.01) | 3.85 |
| Eswatini | 437.74(104.01–1081.33) | 33.86(8.14–83.16) | -0.05(-1.03–0.95) | 10.68 |  | 2.82(0.67–6.98) | 0.22(0.05–0.54) | 4.68(3.82–5.54) | 0.07 |
| Ethiopia | 6761.88(1301.33–22695.3) | 8.11(1.54–27.51) | 2.29(1.78–2.81) | 3.19 |  | 43.62(8.4–146.43) | 0.05(0.01–0.18) | 6.97(6.19–7.76) | 0.02 |
| Fiji | 0.54(0.05–2.24) | 0.06(0.01–0.26) | 4.61(2.29–6.97) | 0.19 |  | 0.05(0–0.2) | 0.01(0–0.02) | 9.62(6.87–12.44) | 0.02 |
| Finland | 8.6(2.79–21.9) | 0.14(0.05–0.36) | 1.73(1.13–2.34) | 3.30 |  | 1.08(0.35–2.75) | 0.02(0.01–0.05) | 6.79(5.88–7.72) | 0.41 |
| France | 83.05(29.6–180.98) | 0.12(0.04–0.27) | 0.06(-0.1–0.22) | 1.90 |  | 10.44(3.72–22.75) | 0.02(0.01–0.03) | 5.08(4.87–5.3) | 0.24 |
| Gabon | 116.92(15.22–427.76) | 7.33(0.96–26.57) | 0.79(0.22–1.36) | 3.06 |  | 0.75(0.1–2.76) | 0.05(0.01–0.17) | 5.41(4.57–6.26) | 0.02 |
| Gambia | 87.78(11.49–296.78) | 5.36(0.69–17.69) | -5.03(-5.71–-4.33) | 2.26 |  | 0.57(0.07–1.91) | 0.03(0–0.11) | -0.45(-0.92–0.01) | 0.01 |
| Georgia | 392.57(225.7–627.5) | 9.96(5.71–15.71) | -1.19(-1.62–-0.75) | 18.12 |  | 86.02(49.46–137.49) | 2.18(1.25–3.44) | 4.16(3.46–4.87) | 3.97 |
| Germany | 188.83(74.79–389.4) | 0.24(0.09–0.49) | 7.88(4.75–11.11) | 3.81 |  | 23.74(9.4–48.94) | 0.03(0.01–0.06) | 13.21(9.64–16.91) | 0.48 |
| Ghana | 1346.05(205.66–3934.35) | 5.1(0.77–14.71) | -1.73(-2.29–-1.17) | 2.55 |  | 8.68(1.33–25.39) | 0.03(0–0.09) | 2.88(2.22–3.54) | 0.02 |
| Greece | 13.51(2.35–41.89) | 0.12(0.02–0.36) | -8.91(-10.34–-7.47) | 3.00 |  | 1.7(0.29–5.27) | 0.01(0–0.05) | -3.89(-5.1–-2.66) | 0.38 |
| Greenland | 0.54(0.08–1.87) | 0.84(0.12–2.92) | 2(0.03–4) | 2.02 |  | 0.07(0.01–0.23) | 0.11(0.02–0.37) | 7.05(4.74–9.41) | 0.25 |
| Grenada | 0.1(0.01–0.47) | 0.09(0.01–0.44) | 5.14(3.26–7.05) | 0.79 |  | 0.01(0–0.04) | 0.01(0–0.03) | 10.43(8.13–12.78) | 0.06 |
| Guam | 0.24(0.03–0.86) | 0.14(0.02–0.49) | -15.64(-17.64–-13.59) | 0.48 |  | 0.02(0–0.08) | 0.01(0–0.04) | -11.42(-13.4–-9.39) | 0.04 |
| Guatemala | 65.44(9.7–203.78) | 0.42(0.06–1.31) | -2.71(-3.07–-2.34) | 2.49 |  | 5.17(0.77–16.09) | 0.03(0–0.1) | 2.07(1.91–2.23) | 0.20 |
| Guinea | 375.09(50.54–1207.6) | 3.89(0.53–12.75) | -3.24(-3.85–-2.63) | 2.15 |  | 2.42(0.33–7.79) | 0.03(0–0.08) | 1.39(0.97–1.81) | 0.01 |
| Guinea-Bissau | 40.4(6.09–135.18) | 2.81(0.43–9.14) | -4.39(-5.44–-3.33) | 1.80 |  | 0.26(0.04–0.87) | 0.02(0–0.06) | 0.11(-0.75–0.99) | 0.01 |
| Guyana | 2.54(0.24–13.39) | 0.33(0.03–1.7) | 4.67(0.96–8.51) | 0.78 |  | 0.2(0.02–1.06) | 0.03(0–0.13) | 9.69(5.41–14.15) | 0.06 |
| Haiti | 35.06(3.62–134.24) | 0.3(0.03–1.13) | 4.48(2.62–6.37) | 0.40 |  | 2.77(0.29–10.6) | 0.02(0–0.09) | 9.76(7.5–12.08) | 0.03 |
| Honduras | 62.6(9.93–202.04) | 0.78(0.13–2.49) | -0.33(-0.63–-0.02) | 2.47 |  | 4.94(0.78–15.95) | 0.06(0.01–0.2) | 4.53(4.03–5.03) | 0.19 |
| Hungary | 8.95(1.64–30.17) | 0.07(0.01–0.24) | -17.41(-21.18–-13.46) | 1.35 |  | 1.96(0.36–6.61) | 0.02(0–0.05) | -12.48(-16.26–-8.52) | 0.29 |
| Iceland | 0.18(0.03–0.67) | 0.05(0.01–0.19) | -19.8(-22.07–-17.46) | 0.98 |  | 0.02(0–0.08) | 0.01(0–0.02) | -15.42(-17.56–-13.23) | 0.12 |
| India | 219862.9(41522–546672.24) | 16.09(3.02–40.3) | -0.51(-1.04–0.01) | 7.22 |  | 5388.81(1017.68–13398.47) | 0.39(0.07–0.99) | 4.16(3.83–4.49) | 0.18 |
| Indonesia | 1268.69(151.1–4816.6) | 0.53(0.06–2.01) | -12.45(-15.91–-8.84) | 0.38 |  | 115.68(13.78–439.21) | 0.05(0.01–0.18) | -8.15(-11.61–-4.56) | 0.03 |
| Iran (Islamic Republic of) | 182.39(51.51–442.07) | 0.22(0.06–0.53) | -1.37(-1.98–-0.76) | 1.75 |  | 6.44(1.82–15.6) | 0.01(0–0.02) | 3.31(2.79–3.83) | 0.06 |
| Iraq | 364.11(106.83–847.04) | 0.98(0.29–2.28) | -1.55(-1.99–-1.1) | 2.88 |  | 12.85(3.77–29.88) | 0.03(0.01–0.08) | 3.13(2.81–3.45) | 0.10 |
| Ireland | 3.86(0.99–11.03) | 0.08(0.02–0.22) | -6.7(-7.64–-5.74) | 1.39 |  | 0.48(0.12–1.39) | 0.01(0–0.03) | -1.81(-2.59–-1.03) | 0.18 |
| Israel | 16.13(5.24–37.52) | 0.17(0.06–0.4) | -2.63(-2.91–-2.35) | 5.60 |  | 2.03(0.66–4.72) | 0.02(0.01–0.05) | 2.41(2.33–2.5) | 0.70 |
| Italy | 123.24(48.59–249.95) | 0.22(0.09–0.45) | -6.42(-7.85–-4.96) | 2.80 |  | 15.49(6.11–31.42) | 0.03(0.01–0.06) | -1.68(-2.98–-0.37) | 0.35 |
| Jamaica | 1.66(0.26–5.89) | 0.06(0.01–0.2) | 2.16(0.49–3.86) | 1.52 |  | 0.13(0.02–0.47) | 0(0–0.02) | 6.98(4.9–9.1) | 0.12 |
| Japan | 126.1(13.66–533.27) | 0.06(0.01–0.23) | -1.19(-1.8–-0.58) | 0.82 |  | 15.85(1.72–67.05) | 0.01(0–0.03) | 3.9(3.02–4.79) | 0.10 |
| Jordan | 19.44(3.34–66.72) | 0.2(0.03–0.66) | -7.66(-9.22–-6.06) | 4.04 |  | 0.69(0.12–2.35) | 0.01(0–0.02) | -3.38(-4.81–-1.92) | 0.14 |
| Kazakhstan | 2247.54(1194.84–3628.87) | 11.91(6.41–19.22) | -12.19(-13.37–-11) | 20.60 |  | 492.45(261.82–795.08) | 2.61(1.41–4.21) | -7.06(-7.97–-6.15) | 4.51 |
| Kenya | 1770(569.76–4253.24) | 4.21(1.35–9.85) | -1.98(-4.1–0.19) | 1.56 |  | 11.42(3.68–27.44) | 0.03(0.01–0.06) | 2.54(0.57–4.56) | 0.01 |
| Kiribati | 0.65(0.06–2.61) | 0.63(0.06–2.53) | 1.89(0.78–3) | 0.29 |  | 0.06(0.01–0.24) | 0.06(0.01–0.23) | 7.16(5.67–8.67) | 0.03 |
| Kuwait | 13.4(3.55–34.23) | 0.28(0.08–0.71) | -3.04(-3.6–-2.48) | 1.60 |  | 0.47(0.13–1.21) | 0.01(0–0.02) | 1.56(0.73–2.39) | 0.06 |
| Kyrgyzstan | 1369.86(486.77–2539.09) | 22.08(7.84–41) | -2.05(-3.16–-0.93) | 25.46 |  | 300.15(106.63–556.33) | 4.84(1.72–8.98) | 3.22(1.8–4.66) | 5.58 |
| Lao People's Democratic Republic | 34.84(4.01–131.14) | 0.57(0.07–2.13) | -8.67(-11.98–-5.23) | 0.47 |  | 3.18(0.37–11.96) | 0.05(0.01–0.19) | -4.24(-7.47–-0.9) | 0.04 |
| Latvia | 47.3(22.97–84.51) | 2.16(1.05–3.86) | -7.76(-9.37–-6.13) | 9.56 |  | 10.36(5.03–18.51) | 0.47(0.23–0.85) | -2.77(-4.29–-1.23) | 2.10 |
| Lebanon | 11.56(1.44–48.83) | 0.22(0.03–0.93) | -0.75(-1.07–-0.43) | 1.83 |  | 0.41(0.05–1.72) | 0.01(0–0.03) | 3.99(3.43–4.56) | 0.06 |
| Lesotho | 435.16(147.53–948.21) | 19.1(6.59–41.85) | 2.12(1.26–2.99) | 4.48 |  | 2.81(0.95–6.12) | 0.12(0.04–0.27) | 6.85(6.16–7.54) | 0.03 |
| Liberia | 120.36(14.33–488.53) | 3.26(0.38–13.13) | -3.09(-3.9–-2.28) | 1.93 |  | 0.78(0.09–3.15) | 0.02(0–0.08) | 1.46(0.88–2.03) | 0.01 |
| Libya | 19.82(2.19–71.73) | 0.29(0.03–1.09) | 0.01(-0.71–0.74) | 1.95 |  | 0.7(0.08–2.53) | 0.01(0–0.04) | 4.92(3.86–5.98) | 0.07 |
| Lithuania | 192.71(111.67–294.51) | 5.91(3.39–9.01) | -3.87(-4.43–-3.32) | 17.06 |  | 42.23(24.46–64.54) | 1.29(0.74–1.97) | 1.38(1.03–1.74) | 3.74 |
| Luxembourg | 0.97(0.14–3.34) | 0.16(0.02–0.56) | -6.66(-7.7–-5.62) | 2.58 |  | 0.12(0.02–0.42) | 0.02(0–0.07) | -1.71(-2.91–-0.49) | 0.32 |
| Madagascar | 1941.99(290.65–5848.27) | 9.43(1.43–28.17) | 6.33(4.27–8.44) | 2.65 |  | 12.53(1.87–37.73) | 0.06(0.01–0.18) | 11.13(8.73–13.58) | 0.02 |
| Malawi | 794.33(152.17–2294.53) | 5.02(1–15.05) | 6.6(6.15–7.04) | 2.03 |  | 5.12(0.98–14.8) | 0.03(0.01–0.1) | 11.65(11.03–12.27) | 0.01 |
| Malaysia | 250.41(60.11–684.49) | 0.8(0.19–2.19) | -3.49(-6.2–-0.7) | 0.83 |  | 22.83(5.48–62.44) | 0.07(0.02–0.2) | 1.19(-1.37–3.82) | 0.08 |
| Maldives | 0.61(0.07–2.51) | 0.13(0.01–0.55) | -10.87(-14.54–-7.05) | 0.43 |  | 0.06(0.01–0.23) | 0.01(0–0.05) | -6.48(-10.14–-2.67) | 0.04 |
| Mali | 688.23(85.7–2050.36) | 4.32(0.54–13.19) | -2.86(-3.44–-2.28) | 2.68 |  | 4.44(0.55–13.23) | 0.03(0–0.09) | 1.74(1.38–2.11) | 0.02 |
| Malta | 0.27(0.04–1.01) | 0.07(0.01–0.25) | -13.86(-14.76–-12.95) | 0.77 |  | 0.03(0–0.13) | 0.01(0–0.03) | -9.37(-10.17–-8.55) | 0.10 |
| Marshall Islands | 0.62(0.1–2.04) | 1.2(0.2–3.97) | -11.6(-14.04–-9.1) | 1.04 |  | 0.06(0.01–0.19) | 0.11(0.02–0.36) | -6.82(-9.13–-4.45) | 0.09 |
| Mauritania | 62.21(7.44–195.75) | 1.98(0.24–6.3) | -5.63(-6.55–-4.7) | 2.28 |  | 0.4(0.05–1.26) | 0.01(0–0.04) | -1.21(-1.95–-0.46) | 0.01 |
| Mauritius | 0.76(0.18–2.25) | 0.05(0.01–0.16) | -12.99(-15.77–-10.12) | 0.49 |  | 0.07(0.02–0.2) | 0(0–0.01) | -8.3(-10.97–-5.55) | 0.04 |
| Mexico | 610.47(119.45–1818.94) | 0.48(0.09–1.43) | 1.89(0.64–3.15) | 3.05 |  | 48.21(9.44–143.66) | 0.04(0.01–0.11) | 7.04(5.87–8.22) | 0.24 |
| Micronesia (Federated States of) | 0.19(0.01–0.91) | 0.2(0.01–0.94) | 0.83(-0.25–1.93) | 0.31 |  | 0.02(0–0.08) | 0.02(0–0.09) | 5.98(4.54–7.43) | 0.03 |
| Monaco | 0.05(0.01–0.19) | 0.12(0.01–0.45) | -2.95(-3.32–-2.58) | 1.25 |  | 0.01(0–0.02) | 0.02(0–0.06) | 2.14(1.95–2.33) | 0.16 |
| Mongolia | 312.81(61.47–871.46) | 8.92(1.76–24.91) | 2.75(1.1–4.43) | 9.71 |  | 68.54(13.47–190.96) | 1.95(0.38–5.46) | 8.25(6.25–10.29) | 2.13 |
| Montenegro | 0.4(0.07–1.29) | 0.05(0.01–0.17) | -5.96(-7.18–-4.72) | 0.93 |  | 0.09(0.01–0.28) | 0.01(0–0.04) | -0.84(-2.28–0.62) | 0.20 |
| Morocco | 570.49(164.11–1349.66) | 1.6(0.46–3.79) | 0.32(-0.17–0.81) | 2.01 |  | 20.14(5.79–47.6) | 0.06(0.02–0.13) | 5.18(4.62–5.75) | 0.07 |
| Mozambique | 3607.32(1165.36–7890.27) | 15.73(5.1–33.65) | 1.24(0.69–1.79) | 5.16 |  | 23.27(7.52–50.91) | 0.1(0.03–0.22) | 5.88(5.16–6.61) | 0.03 |
| Myanmar | 5755.86(2005.38–12002.29) | 10.81(3.81–22.49) | -1.56(-1.85–-1.28) | 5.72 |  | 524.82(182.85–1094.62) | 0.99(0.35–2.05) | 3.37(2.92–3.82) | 0.52 |
| Namibia | 469.66(188.66–916.24) | 18.7(7.56–36.21) | -1.06(-2.06–-0.05) | 6.02 |  | 3.03(1.22–5.91) | 0.12(0.05–0.23) | 3.56(2.75–4.38) | 0.04 |
| Nauru | 0.02(0–0.07) | 0.18(0.02–0.81) | -0.56(-1.24–0.12) | 0.29 |  | 0(0–0.01) | 0.02(0–0.07) | 4.55(3.53–5.57) | 0.03 |
| Nepal | 2178.93(528.8–5773.98) | 7.91(1.94–20.76) | -0.63(-1.18–-0.09) | 4.70 |  | 53.4(12.97–141.53) | 0.19(0.05–0.51) | 4.17(3.74–4.6) | 0.12 |
| Netherlands | 15.1(5.8–32.85) | 0.09(0.03–0.2) | -3.34(-3.66–-3.03) | 1.94 |  | 1.9(0.73–4.13) | 0.01(0–0.02) | 1.67(1.3–2.04) | 0.24 |
| New Zealand | 8.49(1.98–25.16) | 0.19(0.04–0.57) | 0.47(-1.9–2.9) | 1.74 |  | 1.07(0.25–3.16) | 0.02(0.01–0.07) | 5.32(2.49–8.23) | 0.22 |
| Nicaragua | 25.42(4.33–71.51) | 0.43(0.07–1.24) | -1.22(-1.86–-0.57) | 1.83 |  | 2.01(0.34–5.65) | 0.03(0.01–0.1) | 3.61(3.17–4.05) | 0.14 |
| Niger | 611.55(87.34–1945.11) | 3.86(0.55–12.14) | -3.07(-3.69–-2.44) | 2.39 |  | 3.95(0.56–12.55) | 0.02(0–0.08) | 1.4(0.97–1.84) | 0.02 |
| Nigeria | 11962.95(2710.21–33147.59) | 7.68(1.73–20.97) | -2.79(-3.36–-2.23) | 4.15 |  | 77.18(17.48–213.85) | 0.05(0.01–0.14) | 1.81(1.42–2.21) | 0.03 |
| Niue | 0(0–0.01) | 0.1(0.01–0.43) | 0.05(-0.28–0.38) | 0.27 |  | 0(0–0) | 0.01(0–0.04) | 5.17(4.51–5.84) | 0.02 |
| North Macedonia | 3.68(1.2–8.94) | 0.14(0.05–0.34) | -8.47(-8.82–-8.13) | 1.33 |  | 0.81(0.26–1.96) | 0.03(0.01–0.08) | -3.3(-3.53–-3.07) | 0.29 |
| Northern Mariana Islands | 0.19(0.03–0.68) | 0.39(0.06–1.37) | -2.02(-5.73–1.84) | 1.06 |  | 0.02(0–0.06) | 0.04(0.01–0.12) | 2.8(-1.41–7.2) | 0.10 |
| Norway | 7.7(2.08–20.15) | 0.14(0.04–0.37) | -6.81(-7.67–-5.95) | 2.59 |  | 0.97(0.26–2.53) | 0.02(0–0.05) | -1.76(-2.35–-1.17) | 0.33 |
| Oman | 11.32(2.46–32.49) | 0.28(0.06–0.76) | -3.26(-4.61–-1.89) | 1.88 |  | 0.4(0.09–1.15) | 0.01(0–0.03) | 1.4(-0.26–3.08) | 0.07 |
| Pakistan | 27894.46(8879.91–67661.29) | 14.09(4.48–33.72) | 0.61(0.49–0.73) | 5.49 |  | 683.68(217.62–1658.23) | 0.35(0.11–0.83) | 5.33(5.01–5.64) | 0.13 |
| Palau | 0.03(0–0.11) | 0.12(0.01–0.53) | 1.19(0.5–1.88) | 0.31 |  | 0(0–0.01) | 0.01(0–0.05) | 6.4(5.34–7.46) | 0.03 |
| Palestine | 5.66(0.71–23.48) | 0.14(0.02–0.58) | 2.08(0.89–3.28) | 2.12 |  | 0.2(0.02–0.83) | 0(0–0.02) | 6.98(5.4–8.59) | 0.07 |
| Panama | 31.37(3.91–118.51) | 0.75(0.09–2.84) | -1.86(-2.05–-1.67) | 2.31 |  | 2.48(0.31–9.36) | 0.06(0.01–0.22) | 3(2.57–3.42) | 0.18 |
| Papua New Guinea | 398.88(146.03–915.62) | 4.66(1.72–10.59) | 10.91(4.8–17.37) | 3.81 |  | 36.37(13.31–83.47) | 0.43(0.16–0.97) | 15.93(9.13–23.14) | 0.35 |
| Paraguay | 69.6(8.16–228.67) | 1.02(0.12–3.31) | 2.85(2.27–3.43) | 3.34 |  | 5.5(0.65–18.06) | 0.08(0.01–0.26) | 8(7.15–8.85) | 0.26 |
| Peru | 1937.03(973.22–3576.87) | 5.58(2.8–10.31) | -5.23(-8.28–-2.07) | 7.45 |  | 152.98(76.85–282.41) | 0.44(0.22–0.81) | -0.66(-3.6–2.37) | 0.59 |
| Philippines | 9159.48(2581.97–21465.24) | 9.02(2.55–21.3) | 5.86(4.89–6.84) | 2.80 |  | 835.17(235.41–1957.29) | 0.82(0.23–1.94) | 11.07(9.86–12.29) | 0.26 |
| Poland | 49.63(18.43–114.8) | 0.1(0.04–0.22) | -5.19(-6.85–-3.51) | 0.80 |  | 10.87(4.04–25.15) | 0.02(0.01–0.05) | -0.14(-1.64–1.38) | 0.18 |
| Portugal | 20.03(4.52–53.09) | 0.17(0.04–0.45) | -3.96(-5.39–-2.51) | 1.44 |  | 2.52(0.57–6.68) | 0.02(0–0.06) | 0.88(-0.4–2.17) | 0.18 |
| Puerto Rico | 1.52(0.31–4.58) | 0.04(0.01–0.11) | -8.91(-9.64–-8.17) | 1.35 |  | 0.12(0.02–0.36) | 0(0–0.01) | -4.33(-5.17–-3.48) | 0.11 |
| Qatar | 7.18(1.47–22.6) | 0.26(0.05–0.86) | -1.08(-1.39–-0.77) | 2.01 |  | 0.25(0.05–0.8) | 0.01(0–0.03) | 3.66(3.47–3.86) | 0.07 |
| Republic of Korea | 425.45(62.22–1637.04) | 0.57(0.08–2.25) | -5.28(-6.04–-4.52) | 1.43 |  | 53.49(7.82–205.84) | 0.07(0.01–0.28) | -0.49(-1.08–0.11) | 0.18 |
| Republic of Moldova | 950.4(643.71–1322.69) | 22.02(14.94–30.59) | -1.49(-2.25–-0.73) | 37.87 |  | 208.24(141.04–289.8) | 4.83(3.27–6.7) | 3.83(2.78–4.88) | 8.30 |
| Romania | 291.5(118.22–588.13) | 1.29(0.53–2.57) | -1.76(-3.83–0.35) | 3.31 |  | 63.87(25.89–128.88) | 0.28(0.12–0.56) | 3.39(0.91–5.93) | 0.73 |
| Russian Federation | 30972.67(16222.23–49252.93) | 18.75(9.85–29.77) | -7.08(-9.02–-5.1) | 26.14 |  | 6786.34(3554.28–10791.91) | 4.11(2.16–6.52) | -1.77(-3.58–0.08) | 5.73 |
| Rwanda | 663.89(259.22–1395.38) | 6.19(2.41–12.77) | 0.69(-0.52–1.91) | 3.00 |  | 4.28(1.67–9) | 0.04(0.02–0.08) | 5.3(4.29–6.32) | 0.02 |
| Saint Kitts and Nevis | 0.07(0.01–0.32) | 0.11(0.01–0.48) | 4.32(1.89–6.81) | 0.55 |  | 0.01(0–0.03) | 0.01(0–0.04) | 9.58(6.69–12.54) | 0.04 |
| Saint Lucia | 0.11(0.01–0.45) | 0.06(0.01–0.23) | 1.1(0.1–2.1) | 0.46 |  | 0.01(0–0.04) | 0(0–0.02) | 6.2(4.82–7.59) | 0.04 |
| Saint Vincent and the Grenadines | 0.11(0.01–0.42) | 0.09(0.01–0.34) | 1.6(0.37–2.85) | 0.59 |  | 0.01(0–0.03) | 0.01(0–0.03) | 6.69(5.04–8.36) | 0.05 |
| Samoa | 0.07(0.01–0.23) | 0.03(0–0.12) | -4.95(-6.67–-3.19) | 0.07 |  | 0.01(0–0.02) | 0(0–0.01) | -0.37(-1.88–1.16) | 0.01 |
| San Marino | 0.02(0–0.06) | 0.05(0.01–0.17) | -4.47(-5.52–-3.41) | 1.15 |  | 0(0–0.01) | 0.01(0–0.02) | 0.65(-0.2–1.51) | 0.15 |
| Sao Tome and Principe | 4.53(0.58–13.82) | 2.78(0.36–8.58) | -2.68(-3.72–-1.62) | 3.61 |  | 0.03(0–0.09) | 0.02(0–0.06) | 1.75(0.37–3.14) | 0.02 |
| Saudi Arabia | 422.78(78.72–1261.71) | 1.19(0.23–3.49) | -2.39(-3.29–-1.47) | 2.78 |  | 14.92(2.78–44.53) | 0.04(0.01–0.12) | 2.37(1.54–3.2) | 0.10 |
| Senegal | 413.42(124.6–929.24) | 3.53(1.08–7.8) | -1.81(-3.1–-0.51) | 2.23 |  | 2.67(0.8–5.99) | 0.02(0.01–0.05) | 2.62(1.56–3.69) | 0.01 |
| Serbia | 7.72(2.16–20.22) | 0.07(0.02–0.18) | -6.31(-8.08–-4.51) | 0.86 |  | 1.69(0.47–4.43) | 0.02(0–0.04) | -1.08(-2.7–0.57) | 0.19 |
| Seychelles | 0.07(0–0.33) | 0.06(0–0.3) | -6.16(-8.86–-3.39) | 0.22 |  | 0.01(0–0.03) | 0.01(0–0.03) | -1.6(-4.19–1.05) | 0.02 |
| Sierra Leone | 340.72(47.67–1103.58) | 5.3(0.74–16.86) | -2.55(-3.26–-1.84) | 2.21 |  | 2.2(0.31–7.12) | 0.03(0–0.11) | 2(1.5–2.49) | 0.01 |
| Singapore | 23.63(10.07–46.87) | 0.33(0.14–0.67) | -2.79(-4.31–-1.25) | 1.12 |  | 2.97(1.27–5.89) | 0.04(0.02–0.08) | 2.06(0.19–3.97) | 0.14 |
| Slovakia | 2.07(0.42–6.6) | 0.03(0.01–0.09) | -9.3(-10.94–-7.63) | 0.71 |  | 0.45(0.09–1.45) | 0.01(0–0.02) | -4.41(-5.95–-2.84) | 0.16 |
| Slovenia | 0.14(0.02–0.5) | 0(0–0.02) | -18.21(-21.15–-15.16) | 0.11 |  | 0.03(0–0.11) | 0(0–0) | -13.68(-16.67–-10.59) | 0.02 |
| Solomon Islands | 0.74(0.05–2.97) | 0.14(0.01–0.54) | 1.03(0.34–1.73) | 0.31 |  | 0.07(0–0.27) | 0.01(0–0.05) | 6.26(5.22–7.32) | 0.03 |
| Somalia | 4250.54(1163.07–11829.39) | 30.42(8.68–87.51) | -1.62(-2.01–-1.23) | 6.66 |  | 27.42(7.5–76.33) | 0.2(0.06–0.56) | 3.05(2.61–3.49) | 0.04 |
| South Africa | 5851.03(2051.25–13702.4) | 9.98(3.52–23.25) | -1.5(-3.91–0.98) | 2.67 |  | 37.75(13.24–88.38) | 0.06(0.02–0.15) | 3.06(0.76–5.42) | 0.02 |
| South Sudan | 716.11(115.01–2235.71) | 10.85(1.7–33.6) | 0.67(-0.36–1.71) | 3.94 |  | 4.62(0.74–14.42) | 0.07(0.01–0.22) | 5.19(3.81–6.59) | 0.03 |
| Spain | 39.15(5.01–140.62) | 0.08(0.01–0.3) | -5.87(-6.3–-5.44) | 1.19 |  | 4.92(0.63–17.68) | 0.01(0–0.04) | -1.06(-1.33–-0.79) | 0.15 |
| Sri Lanka | 34.13(3.37–131.94) | 0.14(0.01–0.55) | -4.5(-6.24–-2.72) | 0.37 |  | 3.11(0.31–12.03) | 0.01(0–0.05) | 0.5(-1.05–2.08) | 0.03 |
| Sudan | 215.92(24.18–878.28) | 0.64(0.07–2.64) | -2.58(-3.3–-1.86) | 2.04 |  | 7.62(0.85–31.01) | 0.02(0–0.09) | 2.07(1.07–3.08) | 0.07 |
| Suriname | 0.25(0.03–0.88) | 0.04(0.01–0.15) | 1.59(0.26–2.93) | 0.39 |  | 0.02(0–0.07) | 0(0–0.01) | 6.71(4.98–8.46) | 0.03 |
| Sweden | 27.3(9.51–59.13) | 0.27(0.1–0.6) | -1.79(-2.14–-1.44) | 3.11 |  | 3.43(1.2–7.43) | 0.03(0.01–0.08) | 3.28(3.05–3.52) | 0.39 |
| Switzerland | 20.07(8.16–42.07) | 0.24(0.1–0.49) | 6.08(3.61–8.6) | 4.03 |  | 2.52(1.03–5.3) | 0.03(0.01–0.06) | 11.23(8.38–14.17) | 0.51 |
| Syrian Arab Republic | 35.47(4.9–145.79) | 0.25(0.03–1.01) | -3.59(-3.76–-3.43) | 2.64 |  | 1.25(0.17–5.15) | 0.01(0–0.04) | 0.93(0.64–1.23) | 0.09 |
| Taiwan (Province of China) | 246.07(28.53–907.54) | 0.83(0.1–3.05) | -2.46(-3.62–-1.29) | 2.10 |  | 22.44(2.6–82.74) | 0.08(0.01–0.28) | 2.45(1.4–3.5) | 0.19 |
| Tajikistan | 685.44(314.05–1227.84) | 7.61(3.51–13.7) | -0.5(-1.13–0.14) | 14.21 |  | 150.18(68.79–269.05) | 1.67(0.77–3) | 4.8(3.91–5.71) | 3.11 |
| Thailand | 2390.35(659.04–5890.95) | 2.75(0.76–6.83) | -2.03(-3.68–-0.34) | 2.42 |  | 217.95(60.08–537.14) | 0.25(0.07–0.62) | 3.04(1.54–4.55) | 0.22 |
| Timor-Leste | 6.52(0.69–26.41) | 0.61(0.06–2.5) | -6.88(-9.22–-4.49) | 0.48 |  | 0.59(0.06–2.41) | 0.06(0.01–0.23) | -2.36(-4.55–-0.12) | 0.04 |
| Togo | 276.06(41.08–792.63) | 4.36(0.67–12.33) | -3.72(-4.73–-2.69) | 2.43 |  | 1.78(0.26–5.12) | 0.03(0–0.08) | 0.9(0.11–1.7) | 0.02 |
| Tokelau | 0(0–0.01) | 0.12(0.01–0.62) | 0.22(-0.29–0.74) | 0.31 |  | 0(0–0) | 0.01(0–0.06) | 5.37(4.51–6.23) | 0.03 |
| Tonga | 0.09(0.01–0.37) | 0.1(0.01–0.4) | 1.03(0.47–1.6) | 0.29 |  | 0.01(0–0.03) | 0.01(0–0.04) | 6.24(5.33–7.17) | 0.03 |
| Trinidad and Tobago | 0.85(0.1–3.61) | 0.06(0.01–0.23) | 1.62(-0.03–3.29) | 0.56 |  | 0.07(0.01–0.29) | 0(0–0.02) | 6.64(4.53–8.79) | 0.04 |
| Tunisia | 21.99(6.57–53.64) | 0.18(0.05–0.43) | -1.84(-2.63–-1.04) | 1.29 |  | 0.78(0.23–1.89) | 0.01(0–0.02) | 2.81(2.24–3.39) | 0.05 |
| Turkey | 566.59(253.11–1115.31) | 0.64(0.29–1.27) | -2.68(-3.05–-2.32) | 3.80 |  | 20(8.93–39.35) | 0.02(0.01–0.04) | 1.89(1.27–2.52) | 0.13 |
| Turkmenistan | 390.87(143.5–772.45) | 7.68(2.82–15.28) | -0.69(-1.66–0.29) | 16.24 |  | 85.64(31.44–169.25) | 1.68(0.62–3.35) | 4.57(3.29–5.86) | 3.56 |
| Tuvalu | 0.02(0–0.09) | 0.17(0.01–0.77) | 0.4(-0.23–1.04) | 0.31 |  | 0(0–0.01) | 0.02(0–0.07) | 5.55(4.56–6.55) | 0.03 |
| Uganda | 2498.24(526.15–6499.97) | 6.87(1.43–17.81) | 4.28(3.86–4.7) | 3.32 |  | 16.12(3.39–41.95) | 0.04(0.01–0.11) | 9.12(8.82–9.41) | 0.02 |
| Ukraine | 11683.77(6245.43–18445.64) | 23.04(12.3–36.17) | 3.11(0.56–5.72) | 29.52 |  | 2560.01(1368.42–4041.78) | 5.05(2.69–7.93) | 8.52(5.57–11.57) | 6.47 |
| United Arab Emirates | 44.11(4.8–169.38) | 0.44(0.05–1.66) | -1.05(-1.43–-0.66) | 2.02 |  | 1.56(0.17–5.98) | 0.02(0–0.06) | 3.71(3.06–4.37) | 0.07 |
| United Kingdom | 75.93(28.12–165.85) | 0.12(0.04–0.27) | -6.43(-6.94–-5.91) | 1.22 |  | 9.55(3.54–20.85) | 0.01(0.01–0.03) | -1.38(-1.96–-0.78) | 0.15 |
| United Republic of Tanzania | 2785.36(502.18–9275.75) | 6.15(1.08–20.89) | 4.59(3.26–5.93) | 2.51 |  | 17.97(3.24–59.87) | 0.04(0.01–0.13) | 9.29(7.67–10.93) | 0.02 |
| United States of America | 112.23(43.85–242.85) | 0.03(0.01–0.06) | -3.78(-4.95–-2.61) | 1.38 |  | 14.11(5.51–30.53) | 0(0–0.01) | 1.22(0.21–2.23) | 0.17 |
| United States Virgin Islands | 0.03(0–0.11) | 0.03(0–0.1) | 1.49(-0.04–3.04) | 0.48 |  | 0(0–0.01) | 0(0–0.01) | 6.56(4.6–8.56) | 0.04 |
| Uruguay | 2.07(0.45–6.52) | 0.06(0.01–0.18) | 2.9(-0.07–5.95) | 0.32 |  | 0.26(0.06–0.82) | 0.01(0–0.02) | 7.77(4.29–11.37) | 0.04 |
| Uzbekistan | 4070.57(1523.26–7706.34) | 13.17(4.93–24.8) | -5.08(-5.38–-4.78) | 23.92 |  | 891.88(333.76–1688.5) | 2.88(1.08–5.43) | 0.24(-0.12–0.6) | 5.24 |
| Vanuatu | 0.31(0.03–1.34) | 0.12(0.01–0.53) | 4.22(1.78–6.72) | 0.18 |  | 0.03(0–0.12) | 0.01(0–0.05) | 9.25(6.41–12.17) | 0.02 |
| Venezuela (Bolivarian Republic of) | 127.14(15.18–429.09) | 0.44(0.05–1.47) | 0.27(-0.03–0.58) | 1.88 |  | 10.04(1.2–33.89) | 0.03(0–0.12) | 5.27(4.71–5.83) | 0.15 |
| Viet Nam | 6062.61(1778.01–14588.71) | 6.03(1.78–14.42) | -3.93(-5.27–-2.57) | 3.92 |  | 552.79(162.12–1330.31) | 0.55(0.16–1.31) | 1.1(-0.04–2.25) | 0.36 |
| Yemen | 180.06(38.26–657.48) | 0.73(0.15–2.58) | -2.47(-3.98–-0.94) | 2.13 |  | 6.36(1.35–23.21) | 0.03(0.01–0.09) | 2.16(0.8–3.53) | 0.08 |
| Zambia | 1523.04(238.13–5268.18) | 9.31(1.49–32.31) | 7.04(5.5–8.61) | 3.81 |  | 9.83(1.54–33.99) | 0.06(0.01–0.21) | 11.91(10.02–13.83) | 0.02 |
| Zimbabwe | 2075.16(301.14–7024.13) | 13.03(1.89–43.37) | -0.99(-1.13–-0.84) | 5.19 |  | 13.39(1.94–45.32) | 0.08(0.01–0.28) | 3.65(3.32–3.99) | 0.03 |

**Abbreviations:** MDR, multidrug-resistant; XDR, extensively drug-resistant; TB, tuberculosis; ASR, age-standardized rate; CI, confidence interval; EAPC, estimated annual percentage change; UI, uncertainty interval.
